# Supplementary material for: Modeling glioblastoma heterogeneity as a dynamic network of cell states
Source: Mol Syst Biol. 2021 Sep 16;17(9):e10105. doi: 10.15252/msb.202010105 (PMC8444284; doi:10.15252/msb.202010105)
Supplement: Supplementary file 5 — Source Data for Figure 3 [file MSB-17-e10105-s001.zip › Figure3A_sourcedata/GSEA_3065/hallmarks_state2.GseaPreranked.1623416269049/HALLMARK_SPERMATOGENESIS.html]

Details for gene set HALLMARK\_SPERMATOGENESIS[GSEA]

|  || Dataset | state2 |
| Phenotype | NoPhenotypeAvailable |
| Upregulated in class | na\_pos |
| GeneSet | HALLMARK\_SPERMATOGENESIS |
| Enrichment Score (ES) | 0.6444038 |
| Normalized Enrichment Score (NES) | 1.9936427 |
| Nominal p-value | 0.0 |
| FDR q-value | 0.0 |
| FWER p-Value | 0.0 |
Table: GSEA Results Summary

  

Fig 1: Enrichment plot: HALLMARK\_SPERMATOGENESIS      
 Profile of the Running ES Score & Positions of GeneSet Members on the Rank Ordered List

  

| PROBE | GENE SYMBOL | GENE\_TITLE | RANK IN GENE LIST | RANK METRIC SCORE | RUNNING ES | CORE ENRICHMENT || 1 | CDK1 |  |  | 18 | 0.577 | 0.0871 | Yes |
| 2 | DBF4 |  |  | 41 | 0.492 | 0.1608 | Yes |
| 3 | EZH2 |  |  | 70 | 0.417 | 0.2221 | Yes |
| 4 | CDKN3 |  |  | 97 | 0.376 | 0.2773 | Yes |
| 5 | RFC4 |  |  | 126 | 0.339 | 0.3267 | Yes |
| 6 | CCNB2 |  |  | 190 | 0.286 | 0.3642 | Yes |
| 7 | KIF2C |  |  | 252 | 0.251 | 0.3965 | Yes |
| 8 | TOPBP1 |  |  | 323 | 0.225 | 0.4237 | Yes |
| 9 | BUB1 |  |  | 335 | 0.222 | 0.4568 | Yes |
| 10 | NCAPH |  |  | 336 | 0.222 | 0.4910 | Yes |
| 11 | AURKA |  |  | 375 | 0.210 | 0.5193 | Yes |
| 12 | PSMG1 |  |  | 428 | 0.193 | 0.5437 | Yes |
| 13 | VDAC3 |  |  | 528 | 0.176 | 0.5603 | Yes |
| 14 | CNIH2 |  |  | 653 | 0.156 | 0.5712 | Yes |
| 15 | PEBP1 |  |  | 668 | 0.154 | 0.5935 | Yes |
| 16 | NEK2 |  |  | 805 | 0.138 | 0.6002 | Yes |
| 17 | TTK |  |  | 831 | 0.135 | 0.6183 | Yes |
| 18 | PARP2 |  |  | 886 | 0.129 | 0.6325 | Yes |
| 19 | TALDO1 |  |  | 954 | 0.123 | 0.6444 | Yes |
| 20 | RPL39L |  |  | 2249 | 0.055 | 0.5150 | No |
| 21 | TSN |  |  | 2459 | 0.049 | 0.5003 | No |
| 22 | ARL4A |  |  | 2510 | 0.047 | 0.5023 | No |
| 23 | MLF1 |  |  | 2541 | 0.046 | 0.5062 | No |
| 24 | GMCL1 |  |  | 2639 | 0.043 | 0.5026 | No |
| 25 | AGFG1 |  |  | 2754 | 0.040 | 0.4966 | No |
| 26 | STRBP |  |  | 2804 | 0.039 | 0.4974 | No |
| 27 | CSNK2A2 |  |  | 2892 | 0.037 | 0.4938 | No |
| 28 | SIRT1 |  |  | 3423 | 0.025 | 0.4412 | No |
| 29 | COIL |  |  | 3483 | 0.024 | 0.4387 | No |
| 30 | ZC3H14 |  |  | 3546 | 0.023 | 0.4356 | No |
| 31 | STAM2 |  |  | 3826 | 0.018 | 0.4087 | No |
| 32 | SPATA6 |  |  | 4032 | 0.015 | 0.3892 | No |
| 33 | NF2 |  |  | 4275 | 0.011 | 0.3650 | No |
| 34 | PHF7 |  |  | 4419 | 0.009 | 0.3511 | No |
| 35 | MAST2 |  |  | 4431 | 0.008 | 0.3512 | No |
| 36 | MTOR |  |  | 4749 | 0.003 | 0.3180 | No |
| 37 | PRKAR2A |  |  | 4807 | 0.003 | 0.3123 | No |
| 38 | RAD17 |  |  | 4833 | 0.002 | 0.3100 | No |
| 39 | CLPB |  |  | 4881 | 0.001 | 0.3052 | No |
| 40 | IDE |  |  | 4954 | 0.000 | 0.2976 | No |
| 41 | PHKG2 |  |  | 5396 | -0.007 | 0.2516 | No |
| 42 | CHFR |  |  | 5644 | -0.011 | 0.2269 | No |
| 43 | GSTM3 |  |  | 5666 | -0.011 | 0.2264 | No |
| 44 | GAD1 |  |  | 5744 | -0.012 | 0.2201 | No |
| 45 | PGS1 |  |  | 5797 | -0.013 | 0.2166 | No |
| 46 | IFT88 |  |  | 5846 | -0.014 | 0.2136 | No |
| 47 | HSPA4L |  |  | 6125 | -0.019 | 0.1868 | No |
| 48 | MLLT10 |  |  | 6351 | -0.023 | 0.1663 | No |
| 49 | TLE4 |  |  | 6872 | -0.033 | 0.1161 | No |
| 50 | NPHP1 |  |  | 7296 | -0.045 | 0.0779 | No |
| 51 | IP6K1 |  |  | 7452 | -0.049 | 0.0689 | No |
| 52 | LPIN1 |  |  | 8050 | -0.069 | 0.0159 | No |
| 53 | BRAF |  |  | 8328 | -0.084 | -0.0006 | No |
| 54 | JAM3 |  |  | 8782 | -0.120 | -0.0304 | No |
| 55 | PIAS2 |  |  | 8839 | -0.127 | -0.0168 | No |
| 56 | IL13RA2 |  |  | 8854 | -0.129 | 0.0017 | No |
| 57 | SLC12A2 |  |  | 8979 | -0.145 | 0.0109 | No |
| 58 | PCSK1N |  |  | 9321 | -0.247 | 0.0127 | No |
Table: GSEA details [plain text format]

  

Fig 2: HALLMARK\_SPERMATOGENESIS: Random ES distribution      
 Gene set null distribution of ES for **HALLMARK\_SPERMATOGENESIS**
